# Regular exercise attenuates alcoholic myopathy in zebrafish by modulating mitochondrial homeostasis

**DOI:** 10.1371/journal.pone.0294700

**Published:** 2023-11-30

**Authors:** Wei Wen, Cheng Guo, Zhanglin Chen, Dong Yang, Danting Zhu, Quwen Jing, Lan Zheng, Chenchen Sun, Changfa Tang

**Affiliations:** 1 Key Laboratory of Physical Fitness and Exercise Rehabilitation of Hunan Province, College of Physical Education, Hunan Normal University, Changsha, China; 2 School of Physical Education, Hunan First Normal University, Changsha, Hunan, China; National Institutes of Health, UNITED STATES

## Abstract

Alcoholic myopathy is caused by chronic consumption of alcohol (ethanol) and is characterized by weakness and atrophy of skeletal muscle. Regular exercise is one of the important ways to prevent or alleviate skeletal muscle myopathy. However, the beneficial effects and the exact mechanisms underlying regular exercise on alcohol myopathy remain unclear. In this study, a model of alcoholic myopathy was established using zebrafish soaked in 0.5% ethanol. Additionally, these zebrafish were intervened to swim for 8 weeks at an exercise intensity of 30% of the absolute critical swimming speed (Ucrit), aiming to explore the beneficial effects and underlying mechanisms of regular exercise on alcoholic myopathy. This study found that regular exercise inhibited protein degradation, improved locomotion ability, and increased muscle fiber cross-sectional area (CSA) in ethanol-treated zebrafish. In addition, regular exercise increases the functional activity of mitochondrial respiratory chain (MRC) complexes and upregulates the expression levels of MRC complexes. Regular exercise can also improve oxidative stress and mitochondrial dynamics in zebrafish skeletal muscle induced by ethanol. Additionally, regular exercise can activate mitochondrial biogenesis and inhibit mitochondrial unfolded protein response (UPRmt). Together, our results suggest regular exercise is an effective intervention strategy to improve mitochondrial homeostasis to attenuate alcoholic myopathy.

## Introduction

Skeletal muscle is about 40% of body weight and is crucial in protein storage and physical movement [1]. Alcohol is the most commonly consumed beverage worldwide, and excessive alcohol consumption can lead to pathological changes in the skeletal muscle, known as alcoholic myopathy [2–4]. Alcoholic myopathy, characterized by skeletal muscle atrophy and loss of muscle strength, occurs in 40% to 60% of chronic alcoholics [5]. However, the methods and mechanisms for treating alcoholic myopathy remain unclear. Therefore, it is crucial to find ways to alleviate or prevent alcoholic myopathy and to study its underlying molecular mechanisms.

Mitochondria generate ATP through oxidative phosphorylation (OXPHOS) of the mitochondrial respiratory chain (MRC), therefore, MRC is critical for skeletal muscle force generation and maintenance of muscle mass [6]. MRC complexes I, II, and IV function was significantly reduced in myotubes treated with ethanol [7]. Furthermore, the activity of MRC complexes I and III was significantly reduced in ethanol-fed mice [8]. Mitochondria are dynamic organelles that require the coordination of multiple processes (including mitochondrial dynamics, mitochondrial biogenesis, and the mitochondrial unfolded protein response) to maintain mitochondrial homeostasis for normal mitochondrial function [9, 10]. The regulation of mitochondrial dynamics is dependent on the processes of mitochondrial fission and fusion, which have an impact on the shape, size, and quantity of mitochondria [11]. Mitochondrial dynamics imbalance and symptoms of alcoholic myopathy are observed in both alcohol-fed Caenorhabditis elegans and rats [12, 13]. Mitochondrial biogenesis is a crucial process for maintaining the quality of mitochondria, as it aids in replacing damaged mitochondria and preserving their function [14]. Peroxisome proliferators γ activated receptor coactivator 1α (PGC1α) is a crucial transcriptional regulator of mitochondrial biogenesis, which coordinately regulates mitochondrial biogenesis by activating nuclear respiratory factor 2 (NRF2) [14]. In mice with alcoholic liver disease, the expression of PGC1α and NRF2 is down-regulated, and the mitochondrial function is impaired [15, 16]. Mitochondrial unfolded protein response (UPRmt) is a stress response mechanism that aids in maintaining mitochondrial homeostasis and cellular function [17]. In the skeletal muscle of men with chronic alcoholism, the mRNA expression of the UPRmt marker was significantly increased, and skeletal muscle protein metabolism was disturbed [18].

Regular exercise is a common approach to improving skeletal muscle myopathy, maintaining mitochondrial homeostasis, and enhancing mitochondrial function [19–21]. Specifically, regular exercise increases mitochondrial cristae density in athletes and the content of MRC complexes I, III, and IV in the skeletal muscle of older adults [22, 23]. In addition, regular exercise can also activate mitochondrial biogenesis and fusion in skeletal muscle [24, 25]. Moreover, regular exercise can improve alcohol-induced decline in liver mitochondrial function and myocardial oxidative damage [26, 27]. However, the effects of regular exercise on alcoholic myopathy are still unclear. In a previous study, we successfully established an alcoholic myopathy model by soaking zebrafish in 0.5% ethanol [5]. Therefore, the objective of this study was to investigate the effects of exercise on alcoholic myopathy and to gain a better understanding of its underlying mechanism.

## Materials and methods

### Animal and treatment

In this study, a total of 75 male AB line zebrafish were selected at the age of 8 months and divided into three groups: control group (CON, *n* = 25), ethanol treatment group (ET, *n* = 25), and ethanol treatment combined with exercise group (ET-E, *n* = 25). The CON zebrafish were raised in tank water, and the ET and the ET-E zebrafish were raised in a 0.5% ethanol solution. The ET-E zebrafish trained 5 days a week, the swimming intensity was 30% Ucrit, and the swimming time was 2 h/day for 8 weeks. The ET and the ET-E were placed in clean water two hours before and after exercise. The water or 0.5% ethanol solution was replaced every 24 hours, and the ethanol concentration of the ethanol solution was monitored daily. To prevent the acute effects of the last exercise session, tissue sampling was performed 60 hours after the last exercise session. Hunan Normal University’s Laboratory Animal Ethics Committee approved this study (No. 2018–046). To reduce pain during sacrifice, zebrafish were anesthetized using tricaine anesthetic. All animal experiments were performed using all possible methods to reduce or minimize potential pain.

### Zebrafish determination of athletic ability and maximum oxygen uptake(MO_2_max)

Ucrit is the highest continuous swimming speed and is an essential indicator of a zebrafish’s ’exercise ability’. Zebrafish Ucrit and MO_2_max measurements were performed in a miniature variable-speed lane respirator (Loligo Systems, Tjele, Denmark). First, the zebrafish’s body length and weight were measured and fasted for 24 hours. Subsequently, adaptive training was performed in the swimming lane of the respirator for 2 hours at a speed of 0.8 (BL/S), and the water speed was gradually increased at a speed increment of 1.35 (BL/S) every 7 min until the zebrafish reached a state of exhaustion. Finally, calculate the critical swimming speed according to the formula Ucrit = Uf+Us×(Tf/Ts), where Uf is the exhaustion swimming speed; Us is the speed increment; Tf is the time for the highest speed increment; Ts is the time interval. To eliminate the influence of zebrafish body length on swimming speed, the relative critical swimming speed (Ucrit-r) is used to calculate the maximum swimming speed of zebrafish, and the calculation formula is Ucrit-r = Ucrit/BL. Maximum oxygen uptake was calculated according to the formula MO_2_ = SMR + aU2 BL + bU_BL_, SMR (standard metabolic rate) represents the minimum oxygen consumption required by zebrafish at rest; U_BL_ represents real-time swimming speed/body length; a and b are constants.

### Hematoxylin and eosin (HE) staining

The zebrafish were anesthetized with tricaine, and the skeletal muscle was quickly removed and fixed in paraformaldehyde for 24 h, and then washed, dehydrated, transparent, paraffin-penetrated, embedded, and sectioned to make paraffin sections with a thickness about 8 μm. Moreover, carry out HE staining. Observe the HE staining results with an imaging system. The CSA of muscle fibers was calculated using ImageJ software.

### Dihydroethidium (DHE) staining

To analyze skeletal muscle sections, DHE (Thermo Fisher Scientific, USA) was freshly prepared and incubated with the sections for 30 minutes at 37°C in the dark. Nuclei were stained with DAPI for further examination. ROS-positive areas were indicated in red. We acquired images using a fluorescence microscope and quantified fluorescence intensity using ImageJ software.

### Transmission electron microscope (TEM) detection

The soaked electron microscope samples were stored at 4°C, fixed with 1% osmic acid, dehydrated, soaked, embedded, and ultra-thin sectioned, and observed and filmed using a transmission electron microscope (Hitachi H2600) at 5,000 to 20,000 magnifications.

### Mitochondrial oxygen consumption test

Mitochondrial respiration parameters in the zebrafish skeletal muscle of each group were measured using a high resolution Oxygraph 2k (O2K) ventilator and processed using DatLab 6.2 software (Oroboros Instruments GmbH, Innsbruck, Austria). First, we took 5 mg of fresh zebrafish skeletal muscle tissue, added 500 ul mitochondrial respiratory fluid, ground it evenly, added it to the respiratory chamber, covered it to ensure no air bubbles, and absorbed the excess liquid on the lid. Once the respiration rate (red line) had stabilized, substrates for mitochondrial complex pyruvate, malate, and glutamate were added to achieve the peak value of complex I. After equilibrating the red line of the respiration rate, add Cytochrome c to verify the integrity of the mitochondrial membrane. After the red line of respiration rate equilibrated, succinate, the substrate of complex II, was added. Maximum oxidative phosphorylation values for complexes I and II were obtained. After the red line of the respiration rate was equilibrated, the uncoupler CCCP was added. The maximum electron transport capacity values for complexes I and II were obtained. After the red line of respiration rate was equilibrated, the complex I inhibitor rotenone was added. We thus obtained the maximum electron transport capacity value for complex II. After stabilizing the red line of the respiration rate, we added antimycin A, which is an inhibitor of complex III. This allowed us to obtain the residual non-mitochondrial respiration value.

### Quantitative real-time PCR

TRIZOL solution (ThermoFisher Scientific, Waltham, MA, USA) was used for RNA extraction from zebrafish muscle. cDNA was synthesized by reverse transcription of 1 ug RNA using a PrimeScript RT Master Mix (Kusatsu, Shiga, Japan). Quantitative real-time PCR used a SYBR Green PCR kit (Takara). Relative mRNA expression was measured using GAPDH as an internal control and calculated by the 2^-ΔΔCT^ method. The primer sequences used are listed in S1 Table.

### Western blotting

The Western blotting protocol was consistent with the one previously described [5]. Information on the antibodies used is presented in S2 Table.

### Data analysis

Statistical analysis was performed using GraphPad Prism 9 software, and statistical differences were determined by ANOVA. Differences were considered significant if the P value was less than 0.05.

## Results

### Regular exercise ameliorates alcoholic myopathy in ET zebrafish

**Fig 1:**
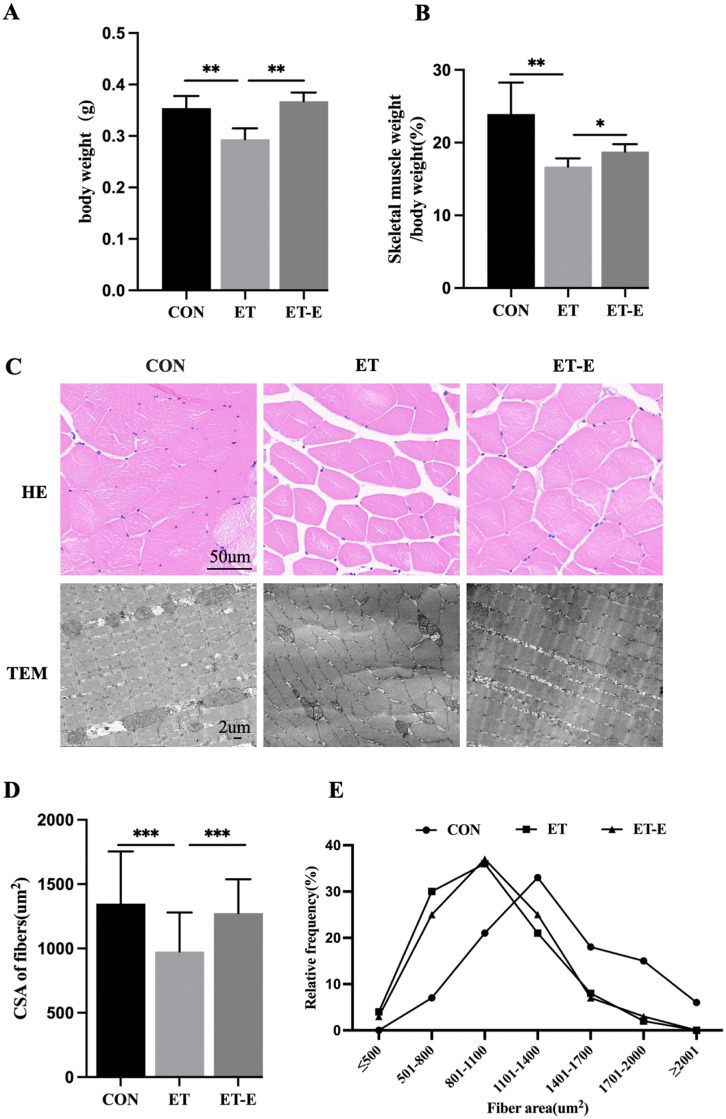
Regular exercise increases ET zebrafish’s muscle fiber CSA and improves skeletal muscle arrangement. (A) Zebrafish body weight (g), *n* = 25. (B) Skeletal muscle mass to body weight (%), *n* = 25. (C) HE staining and TEM imaging of skeletal muscle cross-section. (D) Skeletal muscle fiber’s CSA statistics were produced using ImageJ, *n* = 3. (E) Frequency distribution of muscle fiber area (%). Data are presented as mean ± SD; *P<0.05, **P<0.01, ***P<0.001.

Decreased muscle weight and muscle fibers CSA are skeletal muscle myopathy’s most prominent histopathological features [28, 29]. The body weight and muscle mass/body weight (%) in ET zebrafish were significantly lower than the CON, while regular exercise attenuated ET zebrafish loss of body weight and skeletal muscle (Fig 1A and 1B). HE staining and Transmission electron microscope (TEM) results show that regular exercise decreased muscle fiber spacing, increased the muscle fiber CSA, and improved the disorder of muscle fiber arrangement in ET zebrafish (Fig 1C and 1D). In addition, the proportion of 1101–1400 um^2^ fibers in the CON was the highest, while the distribution of 801–1100 um^2^ fibers was the highest in the ET and ET-E groups (Fig 1E).

### Regular exercise improves the exercise ability of ET zebrafish

**Fig 2:**
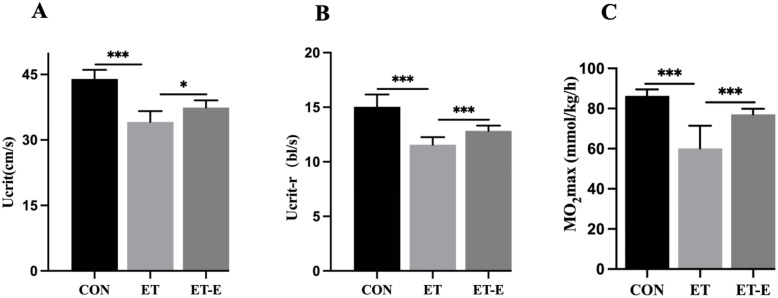
Regular exercise improves locomotion ability in ET zebrafish. (A) Zebrafish Ucrit (cm/s). (B) Zebrafish ucrit-r (bl/s). (C) Zebrafish MO_2_max (mmol/kg/h). Data are presented as mean ± SD; *n* = 25; *P<0.05, ***P<0.001.

The exercise ability of zebrafish was measured by a swimming tunnel respirator. Compared with the CON, the absolute critical swimming speed (Ucrit), relative critical swimming speed (Ucrit-r), and maximal oxygen uptake (MO_2_max) in ET zebrafish were significantly reduced, while regular exercise improved the exercise ability of ET zebrafish (Fig 2A–2C).

### Regular exercise inhibits protein degradation in ET zebrafish

**Fig 3:**
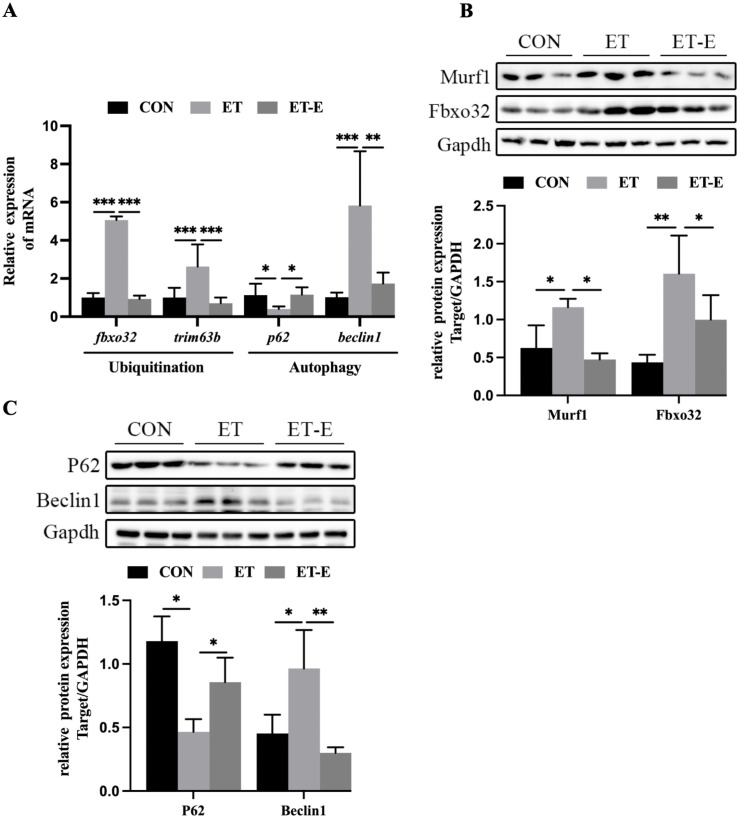
Regular exercise suppresses elevated UPS and ALS in the skeletal muscle of ET zebrafish. (A) mRNA expression of UPS and ALS-related genes in skeletal muscle, *n* = 6. (B) Murf1 and Fbxo32 protein blotting and quantitative analysis, *n* = 3. (C) Beclin1 and P62 protein levels in skeletal muscle, *n* = 3. Data are presented as mean ± SD, *P<0.05, **P<0.01, ***P<0.001.

The proximate cause of alcoholic myopathy is the imbalance between the synthesis and breakdown of protein [30–32]. However, in previous studies, there were no changes in the skeletal muscle protein synthesis signaling pathway (IGF1/PI3K/AKT) in ET zebrafish compared with CON (S1 Fig). The degradation of protein is intricately linked to the ubiquitin-proteasome system (UPS) and autophagy-lysosome system (ALS) [33, 34]. Two E3 ligases: muscle RING finger 1 (MuRF1)/TRIM63, muscle atrophy F-box (MAFbx)/FBXO32, autophagy factor Beclin1, and autophagy receptor P62 are the core factors of UPS and ALS, respectively [28]. The mRNA and protein expression levels of Fbxo32, Murf1, and Beclin1 were upregulated in ET zebrafish skeletal muscle. However, this upregulation was suppressed by regular exercise (Fig 3A–3C). In addition, regular exercise inhibited a decrease in the expression levels of the P62 in ET zebrafish (Fig 3A and 3C).

### Regular exercise improves mitochondrial function in the skeletal muscle of ET zebrafish

**Fig 4:**
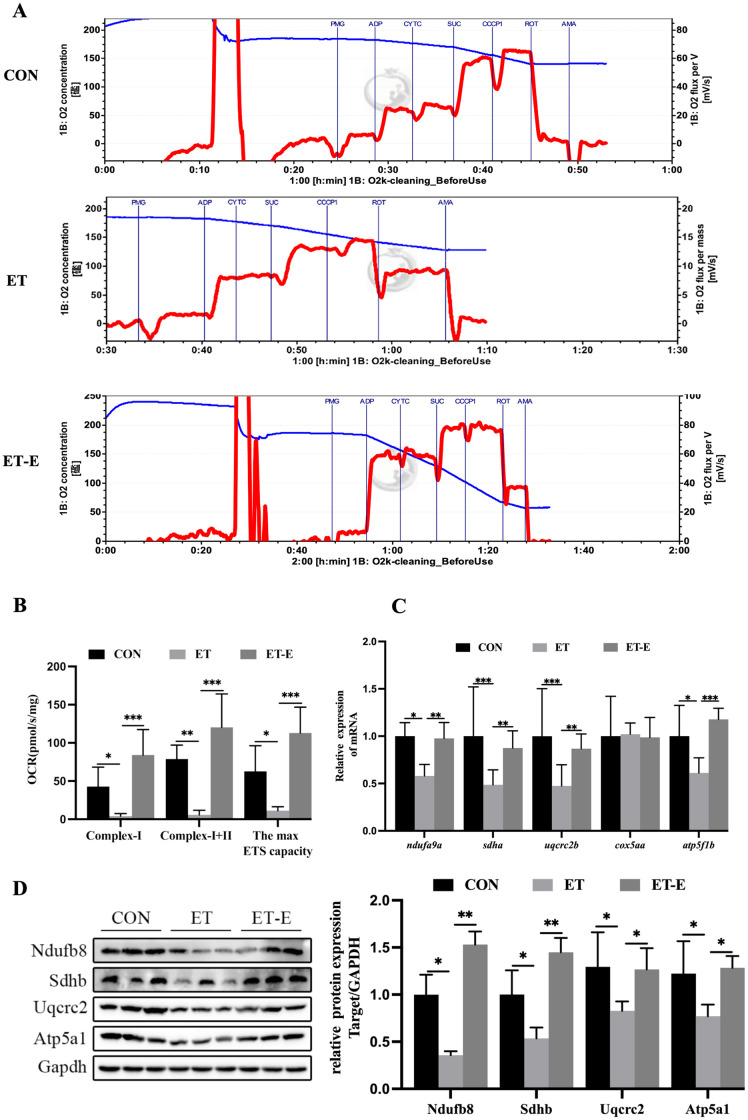
Regular exercise improves activity and content in MRC complexes of skeletal muscle in ET zebrafish. (A) Oxygen consumption curves and mitochondrial oxygen flux as they vary over time following the introduction of different substrates or inhibitors. (B) Oxygen consumption rates (OCR) of mitochondrial complex I, as well as the combined rates of complex I and II, and the maximal activity of the electron transport chain, *n* = *6*. (C) mRNA expression levels of subunits of MRC complexes I (*ndufa9a*), II (*sdha*), III (*uqcrc2b*), IV (*cox5aa*), and V (*atp5f1b*), *n* = 6. (D) Protein levels of subunits of MRC complexes I (Ndufb8), II (Sdhb), III (Uqcrc2), and V (Atp5a1), *n* = 3. Data are presented as mean ± SD; *P<0.05, **P<0.01, ***P<0.001.

To better characterize the effects of regular exercise on mitochondrial function in ET zebrafish, we assessed the activity and content of MRC complexes. The ET zebrafish functional activity of MRC complexes I, I+II, and maximal electron transport chain was reduced compared with CON; however, regular exercise reduced the adverse effects of alcohol on MRC functional activity (Fig 4A and 4B). In addition, regular exercise prevented the decreased mRNA and protein levels of MRC complexes I, II, III, and V in ET zebrafish skeletal muscle (Fig 4C and 4D).

### Regular exercise improves oxidative stress and UPRmt of the skeletal muscle in ET zebrafish

**Fig 5:**
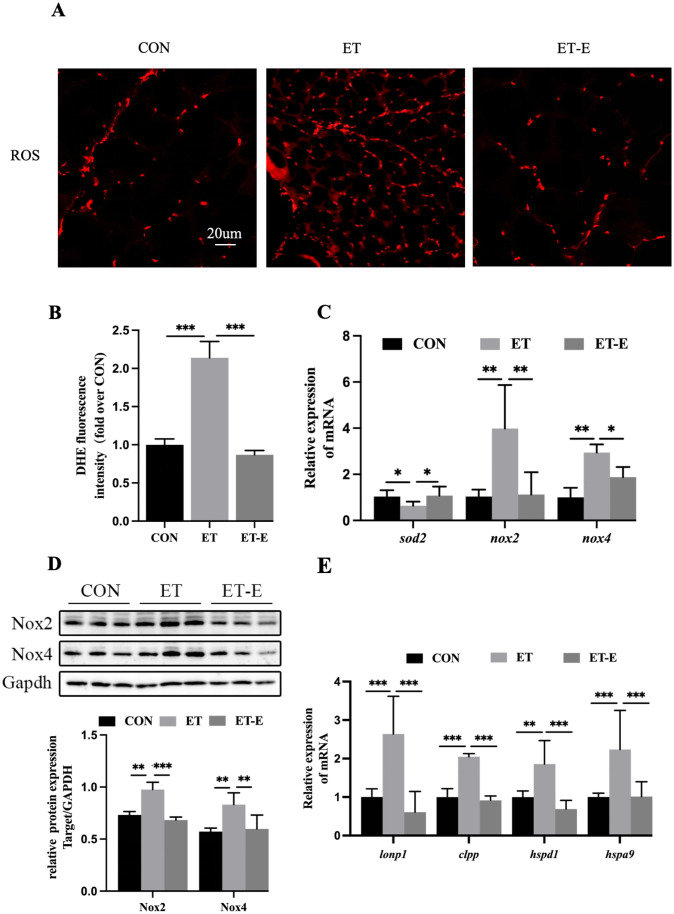
Regular exercise improves ethanol-induced oxidative stress and UPRmt. (A) DHE staining the muscle tissue. (B) Quantitative analysis of skeletal muscle ROS, *n* = 3. (C) The mRNA expression of skeletal muscle oxidase *nox2*, *nox4*, and antioxidant enzyme *sod2*, *n* = 6. (D) Protein levels of oxidase Nox2, Nox4, *n* = 3. (E) UPRmt-related genes mRNA expression levels of *lonp1*, *clpp*, *hspd1*, and *hspa9*, *n* = 6. Data are presented as mean ± SD; *P<0.05, **P<0.01, ***P<0.001.

The impaired function of MRC complexes leads to increases the production of reactive oxygen species (ROS), which in turn causes oxidative stress and damage to the MRC [35]. Assessing the level of ROS in skeletal muscle via DHE staining, we found that the ROS content of ET zebrafish was higher than that of the CON, and regular exercise reversed the increment of ROS in ET zebrafish (Fig 5A and 5B). NADPH oxidase (NOX) is another non-mitochondrial pathway for ROS generation, and superoxide dismutase (SOD) is the main pathway for clearing ROS [36, 37]. The mRNA expression levels of *sod2* were decreased in ET zebrafish, while the mRNA and protein expression levels of Nox2 and Nox4 were significantly increased; however, these ethanol-induced adverse effects were reversed by regular exercise (Fig 5C and 5D). Excessive ROS in vivo interferes with protein folding in mitochondria, triggering the UPRmt, thereby reestablishing mitochondria protein homeostasis [38]. Compared with the CON, the mRNA expressions of *lonp1*, *clpp*, *hspd1*, and *hspa9* in UPRmt-related genes of ET zebrafish skeletal muscle were significantly increased, and regular exercise decreased the mRNA expression of UPRmt-related genes in ET zebrafish (Fig 5E).

### Regular exercise improves mitochondrial morphology and maintains mitochondrial dynamics in ET zebrafish

**Fig 6:**
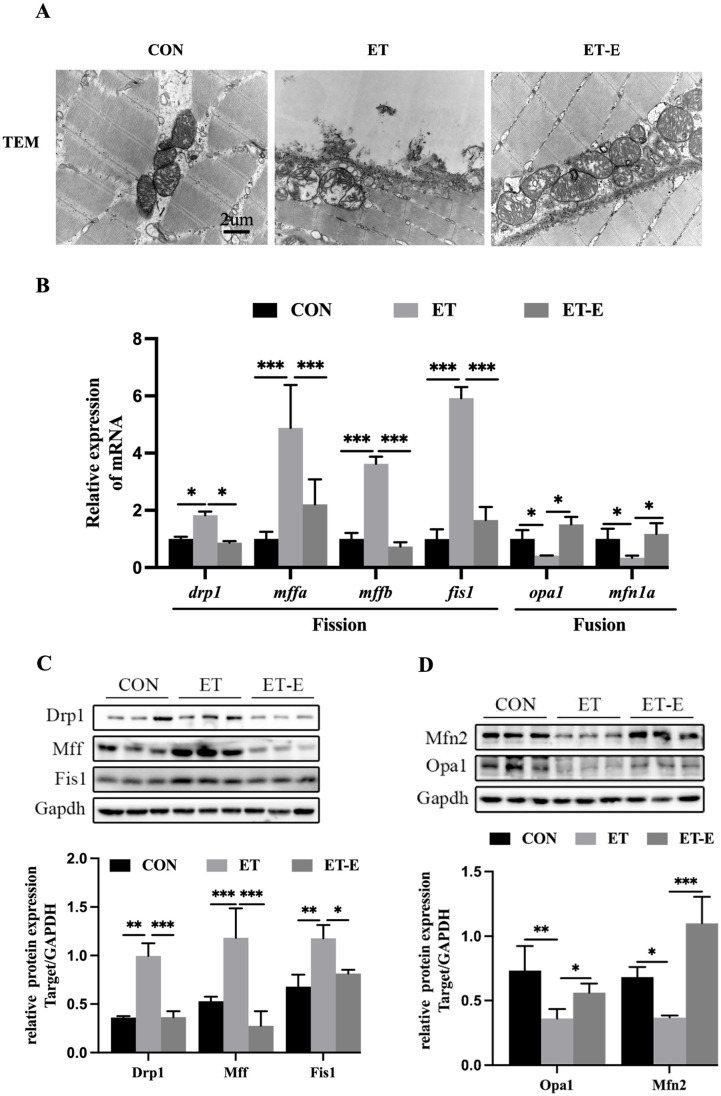
Regular exercise improves mitochondrial morphology and regulates mitochondrial dynamics in ET zebrafish. (A) TEM imaging of muscle tissue mitochondria. (B) mRNA expression levels of genes related to mitochondrial dynamics, *n* = 6. (C) Western blot of mitochondrial fission-related proteins’ banding and quantitative analysis, *n* = 3. (D) Western blot of banding and quantitative analysis of mitochondrial fusion-related proteins, *n* = 3. Data are presented as mean ± SD;*P<0.05, **P<0.01, ***P<0.001.

To obtain a more direct observation of mitochondria, upon examining the mitochondrial morphology of zebrafish skeletal muscle through TEM, it was observed that the mitochondrial cristae were disordered in their arrangement in ET zebrafish. Exercise improved the mitochondrial morphology and enriched the mitochondrial cristae in ET zebrafish (Fig 6A). In addition, We analyzed mitochondrial fission and fusion factors. The results showed that in ET zebrafish skeletal muscle, the mRNA and protein expression of fission factors Drp1, Mff, and Fis1 were increased as compared CON. Regular exercise reversed the increase in levels of mitochondrial fission in ET zebrafish (Fig 6B and 6C). In addition, Regular exercise increased the mRNA expression of fusion factor *opa1* and *mfn1a*, as well as the protein levels of Opa1 and Mfn2 in the skeletal muscle of ET zebrafish (Fig 6B and 6D).

### Regular exercise upregulates mitochondrial biogenesis signaling in ET zebrafish

**Fig 7:**
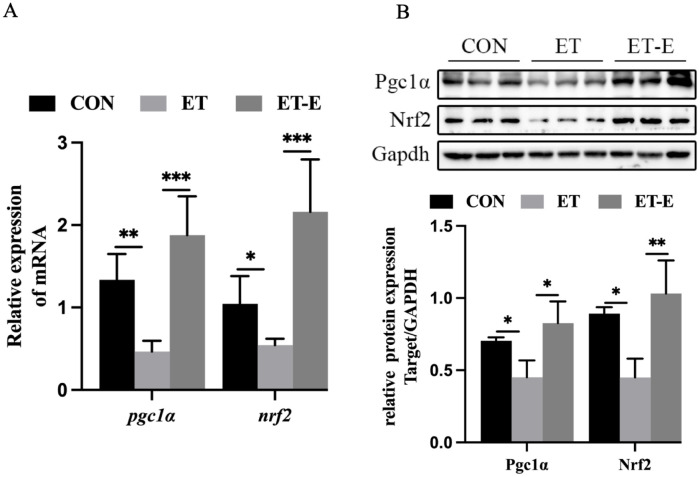
Regular exercise upregulates PGC1α/NRF2 signaling in ET zebrafish. (A) Expression levels of *pgc1α* and *nrf2* mRNA in zebrafish, *n* = 6. (B) Western blot and quantitative analysis of Pgc1α and Nrf2 in skeletal muscle, *n* = 3. Data are presented as mean ± SD; *P<0.05, **P<0.01, ***P<0.001.

The role of the PGC1α/NRF2 signaling pathway in mitochondrial biogenesis is well established [39]. To investigate this, we conducted an analysis of expression levels of Pgc1α and Nrf2. The results showed that both the mRNA and protein levels of Pgc1α and Nrf2 were significantly lower in the ET zebrafish compared to the CON. However, regular exercise increased the expression levels of both Pgc1α and Nrf2 mRNA and protein in ET zebrafish (Fig 7A and 7B).

## Discussion

As the most common skeletal muscle disease, alcoholic myopathy still lacks an effective means of improvement. This study provides a comprehensive experiment that demonstrates how regular exercise can improve zebrafish alcoholic myopathy by regulating mitochondrial homeostasis. Specifically, regular exercise can increase muscle fiber CSA and exercise capacity, reduce oxidative stress, and slow down protein degradation. Moreover, regular exercise can improve mitochondrial dynamic imbalance and mitochondrial structural disorder, slow down UPRmt, increase the activity and content of MRC complexes, and activate mitochondrial biogenesis.

Loss of skeletal muscle mass, decreased muscle fiber CSA, and reduced exercise capacity are common features of alcoholic myopathy [40]. Regular exercise significantly improved body weight, muscle fiber CSA, and exercise capacity in ET zebrafish. This is similar to the results wherein regular exercise improved skeletal muscle atrophy caused by kidney disease [41]. This shows that regular exercise can effectively improve alcoholic myopathy. In cases of skeletal muscle myopathy, including alcoholic myopathy, proteolysis is mainly controlled by two systems: UPS and ALS [42, 43]. In this study, regular exercise inhibited the increased expression of UPS and ALS markers in ET zebrafish skeletal muscle. Illustrates that regular exercise improves alcoholic myopathy by inhibiting UPS and ALS.

The maintenance of skeletal muscle mass and function is highly dependent on the integrity of the MRC [44]. In denervated skeletal muscle atrophy, MRC complexes I, II, and IV activity is significantly reduced [45]. The content of the MRC complex elevates with the increase in human skeletal muscle exercise [22]. Our results demonstrate that regular exercise improves the activity and content of MRC complexes in ET zebrafish. This suggests that regular exercise could potentially aid in improving alcoholic myopathy by restoring the activity and content of MRC complexes. Damage to mitochondrial dynamics may lead to impaired MRC function [46]. Our study found that regular exercise improved both the structure and function of the skeletal muscle mitochondria in ET zebrafish. This improvement in mitochondria function may be attributed to the dynamic changes that occurred.

The reduced activity of MRC complexes I and III led to increased production of mitochondrial ROS, which impaired mitochondrial function [47, 48]. The observed increase in ROS levels in this study may be attributed to damage in the MRC. Excessive accumulation of ROS can further aggravate MRC damage and activate NOX, leading to increased ROS generation through ROS-induced ROS release [49, 50]. We found that ET zebrafish skeletal muscle decreased antioxidant levels and increased oxidation levels, possibly caused by increased mitochondrial ROS generation. According to previous studies, regular exercise has been shown to increase skeletal muscle’s antioxidant capacity and decrease ROS generation and protein degradation [51]. Our findings are in line with these studies, as we observed a significant improvement in the redox balance of ET zebrafish through regular exercise, this was evident through a decrease in ROS content and oxidation capacity and an increase in antioxidant capacity.

The UPRmt is activated in response to a loss of mitochondrial proteostasis [52]. UPRmt activation promotes mitochondrial repair and helps maintain cellular function. Our study found that the markers of UPRmt were also increased in ET zebrafish skeletal muscle, possibly due to damage to the MRC or increased generation of skeletal muscle ROS. However, despite alcohol activating UPRmt, restoration of UPRmt was found to be insufficient in maintaining mitochondrial homeostasis. We also found that regular exercise attenuated ethanol-induced UPRmt in zebrafish. Paradoxically, high-intensity exercise activates UPRmt in the skeletal muscle of aged mice, and this difference may be due to varying exercise intensity [53].

Mitochondrial dynamics are critical for reducing sarcopenia, maintaining the balance of skeletal muscle protein metabolism, and the health of mitochondria [54, 55]. Research has shown that inhibiting mitochondria fission can prevent muscle atrophy, conversely, specific overexpression of the fission factor Drp1 in muscle can result in loss of muscle mass and decreased exercise performance [56]. In addition, alcoholic liver disease patients have reduced circumference and area of skeletal muscle mitochondria, and alcohol also increases the expression of fission factors in epithelial cells [57, 58]. Regular exercise can improve aging to induced sarcopenia by promoting mitochondrial fusion and inhibiting fission [59]. Our research results suggest that ET zebrafish have a shifted mitochondrial dynamics balance towards fission. However, regular exercise can promote mitochondrial fusion, and inhibit mitochondrial fission.

Mitochondrial biogenesis cooperates to maintain mitochondrial homeostasis and muscle mass [60, 61]. However, aging skeletal muscle experiences decreased levels of PGC1α, impaired MRC function, and muscle atrophy [62]. However, increased expression of PGC1α can inhibit the function of UPS and ALS, thus alleviating disuse muscle atrophy [63, 64]. Regular exercise can activate skeletal muscle mitochondrial biogenesis and improve skeletal muscle function and sarcopenic phenotype in aged mice [24, 65]. In this study, regular exercise significantly upregulated PGC1α/NRF2 signaling, indicating that regular exercise can activate mitochondrial biogenesis in ET zebrafish. However, this study still has some limitations. First of all, due to funding and technical reasons, this study only reported that exercise can improve alcoholic myopathy in male zebrafish, and did not conduct detailed verification and analysis of its specific mechanism. Second, we did not explore which muscle fiber types were specifically improved by exercise. Next, we will use omics and CRISPR/Cas9 technology to explore and verify the specific molecular mechanism.

In conclusion, regular exercise improves skeletal muscle oxidative stress and mitochondrial function and maintains mitochondrial homeostasis, inhibiting protein degradation and attenuating ethanol-induced alcoholic myopathy. This provides new targets and ideas for preventing and treating alcoholic myopathy.

## Supporting information

S1 Table List of primers used for RT-qPCR. (XLSX)

S2 Table List of primary antibodies used for Western blotting. (XLSX)

S1 Fig mRNA and protein expression levels of IGF1/PI3K/AKT signaling. (EPS)

S1 Raw images (PDF)
