# Supplementary material for: Regular exercise attenuates alcoholic myopathy in zebrafish by modulating mitochondrial homeostasis
Source: PLoS One. 2023 Nov 30;18(11):e0294700. doi: 10.1371/journal.pone.0294700 (PMC10688687; doi:10.1371/journal.pone.0294700)

Western blot original images of Figure.3

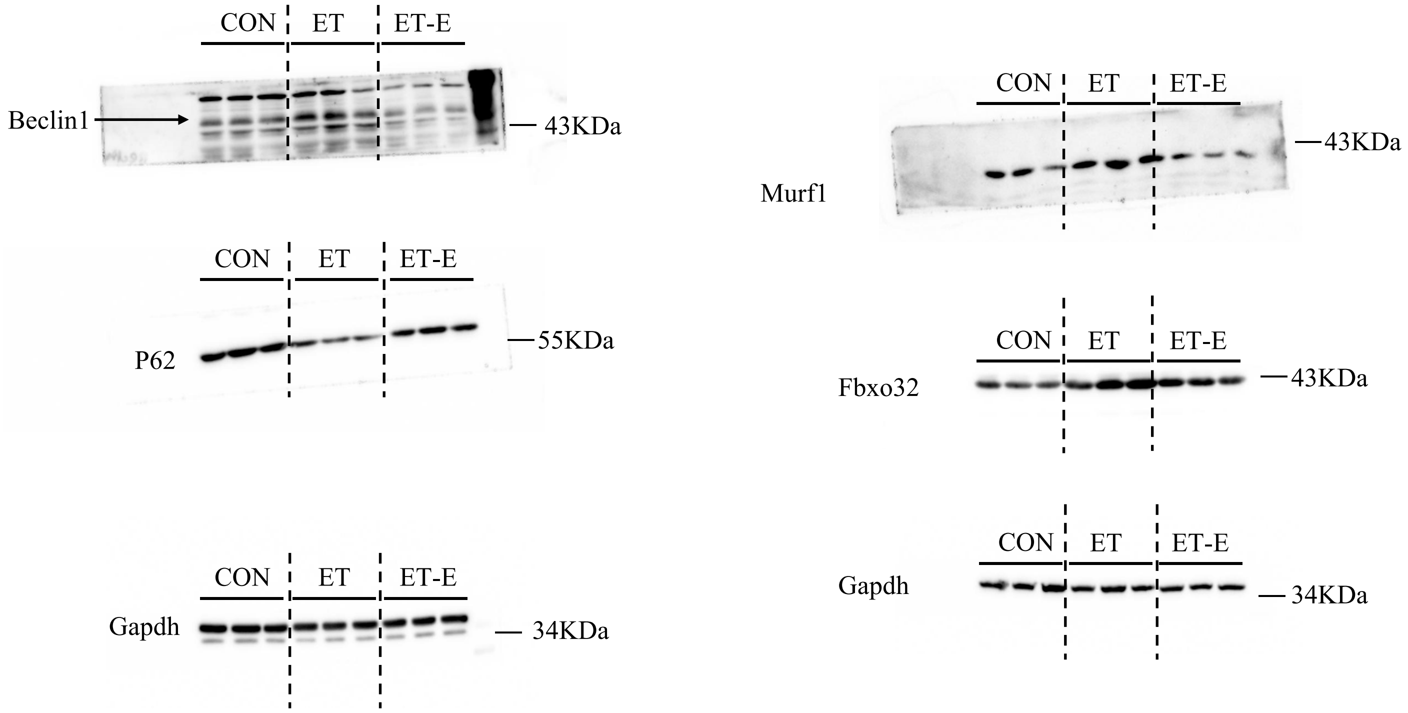

Western blot original images of Figure.4

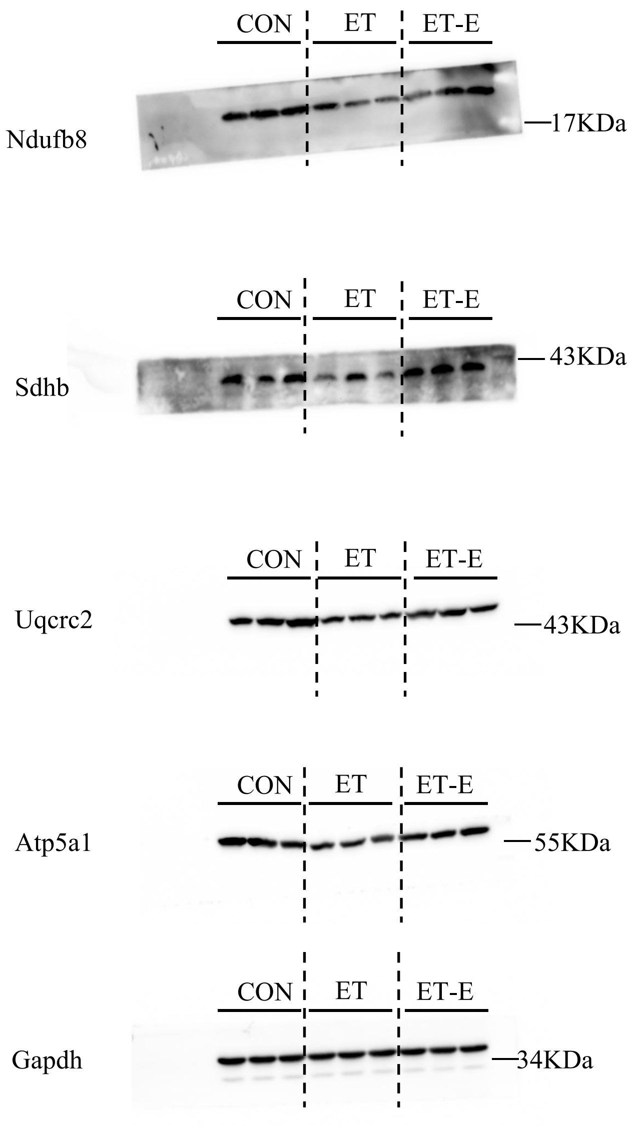

Western blot original images of Figure.5

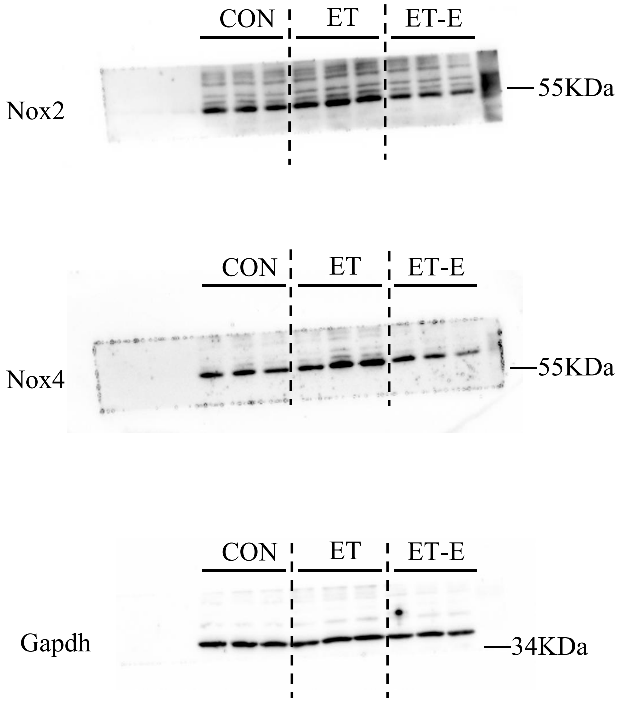

Western blot original images of Figure.6

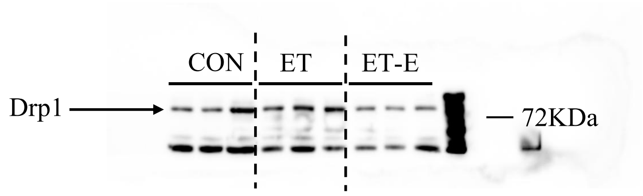

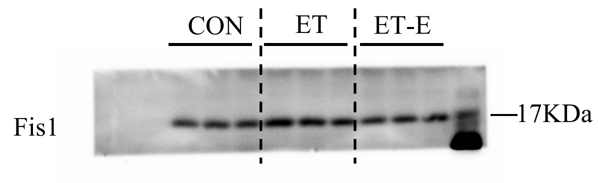

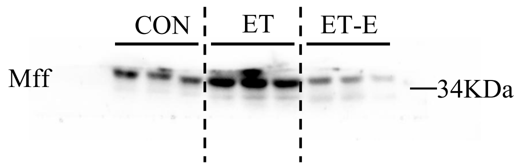

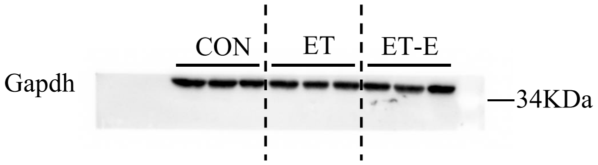

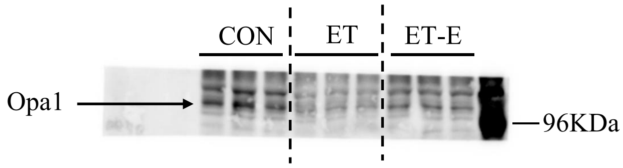

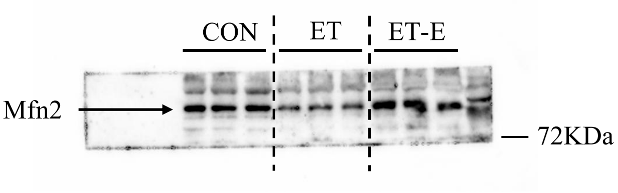

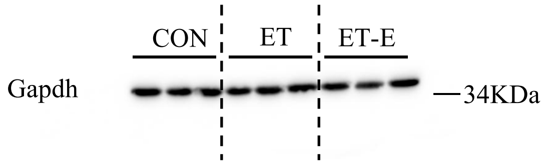

Western blot original images of Figure.7

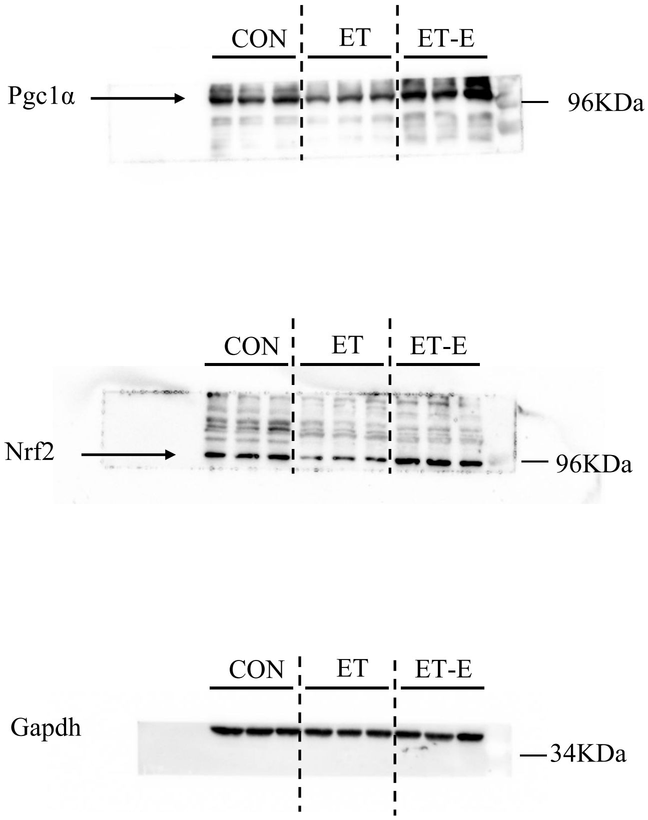

Supplement: S1 Raw images — (PDF) [file pone.0294700.s004.pdf]
